# Pregnancy Incidence and Correlates during the HVTN 503 Phambili HIV Vaccine Trial Conducted among South African Women

**DOI:** 10.1371/journal.pone.0031387

**Published:** 2012-04-19

**Authors:** Mary H. Latka, Katherine Fielding, Glenda E. Gray, Linda-Gail Bekker, Maphoshane Nchabeleng, Koleka Mlisana, Tanya Nielson, Surita Roux, Baningi Mkhize, Matsontso Mathebula, Nivashnee Naicker, Guy de Bruyn, James Kublin, Gavin J. Churchyard, on behalf of the HVTN 503 Phambili study team

**Affiliations:** 1 The Aurum Institute, Johannesburg, South Africa; 2 The London School of Hygiene and Tropical Medicine, London, United Kingdom; 3 Perinatal HIV Research Unit, University of Witwatersrand, Johannesburg, South Africa; 4 Desmond Tutu HIV Foundation, University of Cape Town, Cape Town, South Africa; 5 Medical University of South Africa (Medunsa) HIV Clinical Research Unit, University of Limpopo, Pretoria, South Africa; 6 Centre for AIDS Programme for Research in South Africa (CAPRISA), University of KwaZulu-Natal, Durban, South Africa; 7 Vaccine and Infectious Disease Division, Fred Hutchinson Cancer Research Center, Seattle, Washington, United States of America; Tulane University, United States of America

## Abstract

**Background:**

HIV prevention trials are increasingly being conducted in sub-Saharan Africa. Women at risk for HIV are also at risk of pregnancy. To maximize safety, women agree to avoid pregnancy during trials, yet pregnancies occur. Using data from the HVTN 503/“Phambili” vaccine trial, we report pregnancy incidence during and after the vaccination period and identify factors, measured at screening, associated with incident pregnancy.

**Methods:**

To enrol in the trial, women agreed and were supported to avoid pregnancy until 1 month after their third and final vaccination (“vaccination period”), corresponding to the first 7 months of follow-up. Unsterilized women, pooled across study arms, were analyzed. Poisson regression compared pregnancy rates during and after the vaccination period. Cox proportional hazards regression identified associations with first pregnancy.

**Results:**

Among 352 women (median age 23 yrs; median follow-up 1.5 yrs), pregnancy incidence was 9.6/100 women-years overall and 6.8/100 w-yrs and 11.3/100 w-yrs during and after the vaccination period, respectively [Rate Ratio = 0.60 (0.32–1.14), p = 0.10]. In multivariable analysis, pregnancy was reduced among women who: enrolled at sites providing contraception on-site [HR = 0.43, 95% CI (0.22–0.86)]; entered the trial as injectable contraceptive users [HR = 0.37 (0.21–0.67)] or as consistent condom users (trend) [HR = 0.54 (0.28–1.04)]. Compared with women with a single partner of HIV-unknown status, pregnancy rates were increased among women with: a single partner whose status was HIV-negative [HR = 2.34(1.16–4.73)] and; 2 partners both of HIV-unknown status [HR = 4.42(1.59–12.29)]. Women with 2 more of these risk factors: marijuana use, heavy drinking, or use of either during sex, had increased pregnancy incidence [HR = 2.66 (1.24–5.72)].

**Conclusions:**

It is possible to screen South African women for pregnancy risk at trial entry. Providing injectable contraception for free on-site and supporting consistent condom use may reduce incident pregnancy. Screening should determine the substance use, partnering, and HIV status of both members of the couple for both pregnancy and HIV prevention.

**Trial Registration:**

SA National Health Research Database DOH-27-0207-1539; Clinicaltrials.gov NCT00413725

## Introduction

Clinical trials to test effectiveness of HIV preventive methods are increasingly being conducted in Sub-Saharan Africa where HIV incidence is high. Women at risk for HIV recruited for these trials are also often at high risk of pregnancy, yet are asked to avoid pregnancy whilst on investigational products regardless of the trial phase, as safety to the unborn child is usually unknown. In microbicide trials, women are tested for pregnancy frequently and are taken off study product if they become pregnant. In vaccine trials, women commit to avoiding pregnancy during the vaccination period, and the vaccination schedule is halted if a woman becomes pregnant. Being able to identify women at higher risk for pregnancy at screening may enhance participant safety and minimizes time off study product, which increases trial efficiency [1].

While pregnancy incidence rates were as high as 64/100 woman-years (/100 w-yrs) in early microbicide trials [2], [3] attributed in part to ascertainment bias, rates from recently-completed trials testing microbicides, the diaphragm and an HSV-suppression strategy have been lower, ranging from 4.0–27.1/100 w-yrs [4]–[8]. Nonetheless, pregnancies still occurred despite women’s expressed commitment, and need, to avoid pregnancy during trial participation. Pregnancy risk during vaccine trials is poorly characterized. Of the two vaccine trials in sub-Saharan Africa [9], [10], one reported on pregnancy, and found a cumulative pregnancy incidence of 8.7% overall, but did not report on risk factors [10]. In a large vaccine trial in Thailand, 4.3% of women became pregnant during the 7-month vaccination period, but again, risk factors were not evaluated [11], [12].

Factors associated with pregnancy in HIV prevention trials conducted in sub-Saharan Africa are only now being reported. In an analysis of multiple trials, use of injectable hormonal contraception was associated with reduced pregnancy incidence [8]; while use of oral contraceptive pills (OCPs), method switching and younger age were commonly associated with increased incidence [7], [8], [13]_._ Lack of convenient availability and the perception that available contraceptive services are of low quality have been noted as barriers to contraceptive use among women participating in an African vaccine trial [10]. The role of condoms during trials has been mixed. In one study condoms were associated with reduced pregnancy risk only when condom use was carefully measured [7], while another found that condom use at last sex, but not “condom use” generally, was associated with reduced pregnancy rates [8]. These findings, along with variations in pregnancy rates between trials, suggest that pregnancy risk can be modified. It is particularly important to identify factors that can help African women avoid pregnancy in vaccine trials as the experimental dose cannot be readily withdrawn, yet nothing has been reported on risk factors for incident pregnancy during vaccine trials.

Using data from women who participated in the HVTN 503 “Phambili” trial testing an HIV vaccine, we report pregnancy rates and outcomes during and after the vaccination period, and identify factors reported at screening that were associated with incident pregnancy during this trial. We also evaluated associations of contraception and condom use during the trial with pregnancy incidence as such use may be modified by trial staff through counselling and enhanced access. Findings from this analysis may improve screening and support of women in minimizing pregnancy during future HIV prevention trials in sub-Saharan Africa.

## Methods

### Ethics Statement

This analysis was approved by the ethics boards governing all 5 trial sites, including the University of KwaZulu Natal (2 sites), The University of Cape Town, The University of the Witwatersrand, and the Medical University of South Africa, as well as the London School of Hygiene and Tropical Medicine (statistician, author KF), the Medicines Control Council of South Africa and the Genetically Modified Organism Review Committee of the South African Department of Agriculture.

### Study Design

While these data derive from a randomized trial [14], the study design for this analysis was an observational cohort. Women were pooled across trial arms as the experimental product was found to be ineffective against HIV [14], [15] and was not hypothesized to affect fertility.

### Sample

Women enrolled in HVTN 503 “Phambili”, an HIV vaccine trial, comprised the source population for this analysis [14]. Participants were recruited at five sites in South Africa: Cape Town, Durban, Klerksdorp, Soweto and Pretoria. Phambili was a two-arm, phase IIB, double-blind randomized trial to evaluate the safety and efficacy of a 3 dose-regimen (given at enrolment, 1 and 6 months) of the clade B Merck adenovirus serotype 5 HIV-1 gag/pol/nef vaccine. Men and women aged 18 to 35 were eligible for enrolment. The eligibility criteria for women were being: sexually active in the 6 months prior to screening, in good health with ALT levels less than 2.6 times the upper limit of normal range, sero-negative for HIV1 and 2, and not breastfeeding nor pregnant at enrolment. All women also had to agree to avoid pregnancy during the vaccination period, which encompassed the first 7 months of trial follow up. After screening, non-sterile women also had to agree to consistently use at least 2 forms of contraception: 1 hormonal and 1 barrier method until at least 1 month after her third, and final scheduled, vaccine injection (the “vaccination period”). At enrolment women had to provide documentation of hormonal method use for at least 21 days prior to enrolment, when the first vaccine was injected.

Detailed exclusion criteria are available elsewhere [14], but generally persons were excluded if they were immune deficient or had an auto-immune disease, had ever taken part in an HIV vaccine trial, had recently (5 to 90 days before enrolment) received immunosuppressive therapy, blood or immuoglobin, or other vaccines or allergy treatment. All participants gave written informed consent, and had to demonstrate their understanding of trial concepts prior to enrolment.

### Analytic Sample

Of the 801 persons enrolled, 360 were women, and 352 were at risk for pregnancy at enrolment and included in this analysis (n = 7 women were surgically sterilized before screening; n = 1 was later determined to be pregnant at enrolment).

### Independent Variables

Data on demographics, contraceptive and condom use, sexual behaviour, sexual partners (numbers, HIV status and risk profile), substance use, and history of sexually transmitted infection (STI) were collected via structured, face-to-face interviews by nurses during the screening period (referred to as screening or baseline variables). Screening could take place up to 56 days prior to enrolment, and women had their screening interviews a median of 15 days prior to enrolment. At screening all variables were asked in reference to the six months prior, except contraception and condoms which referred to current use. Use of condoms was asked in two ways: whether currently being used via a single question, and through a series of questions about condom use in the 6 months prior, by partner type and whether condom use with that partner type was consistent or not. Both condom use variables were examined given mixed evidence on the role of condom use in pregnancy prevention; each variable was considered separately in modelling to avoid collinearity.

One site-level variable was considered. Three of the research sites supplied free hormonal contraception (injectables or the pill), throughout the trial. At the remaining sites women were referred to free public sector clinics for injectables or oral contractive pills. Sites were categorized by whether hormonal contraception was available on or off site.

Contraceptive, condom use, and partner data were also collected at 3, 7, 12 and 18 months of follow up, and data from the latter 3 visits were analysed as they provided non-overlapping information (questions referred to “last 6 months”). We analysed current condom use (yes/no), consistent condom use, and hormonal contraception use during the trial. Data on other forms of protection were too infrequent for analysis. Contraceptive use during the trial (time-varying variables) was not independent of use at screening, and therefore the former were not included in multivariable modelling.

### Dependent Variable: Incident Pregnancy

The main outcome variable was first pregnancy during follow up, whether within or outside the vaccination period. Pregnancies were measured in 1 of 2 ways: either via a ß-HCG urine pregnancy test, or by dating the pregnancy from last menstrual period if a woman reported being, or was clinically noted as, pregnant. Of the 48 pregnancies observed, 30 (62%) were confirmed with a urine pregnancy test; 85% (11/13) and 54% (19/35) of pregnancies were confirmed with a urine test during the vaccination period, and post-vaccination period, respectively. Date of incident pregnancy was defined either as 14 days after the last menstrual period (LMP), or if LMP was unknown, then as the estimated date of delivery date minus 266 days.

### Per Protocol Pregnancy Testing, and Pregnancy Prevention Counselling

Vaccinations were scheduled to occur at enrolment and months 1 and 6. All women were counselled and supported to avoid pregnancy during the vaccination period, defined as the period from enrolment (first vaccine) until 1 month after last vaccination – or the first 7 months of follow up for each woman. The protocol specified pregnancy testing before each vaccination was administered, and thus pregnancy testing was routinely done during the vaccination period. After the vaccination period, pregnancy testing was done if indicated or requested by the participant. The differential in pregnancy testing during and after the vaccination period was to avoid administration of the experimental vaccine to a pregnant woman.

### Actual Pregnancy Testing and Pregnancy Prevention Counselling

Enrolment for this trial started 24 January 2007. Enrolment and all vaccinations were unexpectedly halted on 19 September 2007 when it became known, through another trial testing the same product, that the vaccine product under testing was not effective in preventing HIV or reducing early post-infection viral load. The Phambili trial was testing the same product as the Step trial (HVTN 502) [15], which had started several years before and released its findings of no effect, when enrolment for the Phambili trial was in its ninth month. The unanticipated cessation of enrolment and vaccinations for the Phambili trial meant that not all enrolled received the full course of 3 vaccines and the vaccination period–the time when pregnancy was to be avoided–varied in length for each woman depending on when she was enrolled. Additionally after 19 September pregnancy prevention messages varied. One site counselled women to adhere to the initial plan, and to avoid pregnancy until one month after what would have been her third vaccination (avoid for 7 months). Two sites counselled women to avoid pregnancy until 1 month after her last vaccination, whether it was the first, second or third. Two sites counselled women to avoid pregnancy until they were ready to have children. All women were supported and counselled to avoid pregnancy during the vaccination period, and all women received as-needed contraception counselling (and were provided with contraception at sites with that facility) throughout the trial.

### Pregnancy Rates and Outcomes Stratified by Vaccination Period

Given the varied pregnancy prevention messages after vaccinations were stopped, we considered several ways of defining the vaccination period. Upon further examination of the frequency of pregnancy testing, and despite variations in pregnancy counselling, pregnancy testing remained largely in line with the protocol: 87% of women were tested for pregnancy at the end of their initially-scheduled vaccination period, and pregnancy testing became less frequent thereafter. Therefore we defined the vaccination period as the first 7 months of follow up. We calculated pregnancy rates stratified by the vaccination period (during versus after), with the expectation that observed pregnancy rates may have been under-estimated in the post-vaccination period. We show pregnancy outcomes overall and stratified by vaccination period; data were too spare for further statistical analysis.

### Time at Risk

The follow-up period for this analysis spans from 24 January 2007, the first date a vaccination occurred, through 5 May 2009 when data were pulled for this analysis. The enrolment period spanned from 24 January through 18 September 2007. Women contributed person years from first vaccination to first pregnancy defined as the last menstrual period plus 14 days, or the date of her last visit within the follow-up timeframe noted above. For women with multiple pregnancies (3 women had 2 pregnancies) only the first pregnancy was counted.

### Statistical Methods

To describe the enrolled sample, we calculated the frequency of women’s demographic, contraceptive use, sexual behaviour and risk profiles as measured at screening. Overall pregnancy incidence is expressed per 100 women-years with an associated 95% confidence interval. Pregnancy incidence was stratified by socio-demographics, behaviours, partner profile, and vaccination period. Poisson regression was used to compare pregnancy rates during and after the vaccination period. Cox proportional hazards regression was used to assess predictors measured at screening with time to first pregnancy, and to assess the role of contraceptive use during the trial by allowing contraceptive use to be time varying. Given the modest sample size and the need for parsimony during model building, only contraceptive use was considered for the time-varying analysis, and interaction terms to test for joint effects were not evaluated To show joint effects of the identified behavioural risk factors, pregnancy rates stratified by number of risk behaviours (none or one versus two or more) were shown. *A priori* considerations (both condom use measures) or variables with p<0.20 in univariable analysis were considered for multivariable modelling. Following adjustment, any variable with a p-value>0.2 was excluded from the model.

## Results

Women (n = 352) were predominantly young (median 23 years, inter-quartile range (IQR) 20–27) and Black African 98.9% (Table 1). At screening all reported intent to avoid pregnancy during the vaccination period. About two thirds (68.8%) of women were enrolled at a site that provided hormonal contraception. In this analysis, women were followed for a median of 1.5 years (IQR 1.36–1.74), with minimum time at risk of 1 day (for one woman found pregnant at her first vaccination follow up visit) to a maximum of 2.24 years. For this analysis, retention was 100%, 89% and 85% at months 7, 12 and 18, respectively.

**Table 1:** Demographic characteristics and contraceptive use by incident pregnancy.

	Column % (n)	Rate per 100 woman yrs (No. pregnancies/woman yrs)	Unadjusted Rate Ratio (95% CI)	P-value
**Overall pregnancy incidence**	(352)	9.6 (48/501)	–	–
**Age in years**				0.24
18–20	28.7 (101)	11.3 (16/142)	1	
21–25	38.9 (137)	9.9 (19/192)	0.89 (0.46–1.72)	
26–30	20.2 (71)	10.7 (11/103)	0.94 (0.44–2.03)	
31–35	12.2 (43)	3.1 (2/64)	0.28 (0.06–1.23)	
**Race**				0.53
Black	98.9 (348)	9.5 (47/496)	1	
Mixed	1.1 (4)	19.4 (1/5)	2.05 (0.28–14.8)	
**Contraception supplied at site**				
Yes	68.8 (242)	8.3 (29/348)	0.68 (0.38–1.21)	
No	31.3 (110)	12.4 (19/153)	1	0.20
**Contraception use at screening**				
**Consistent condom use in 6 months prior to screening**				
Yes	57.1 (201)	8.6 (25/290)	0.78 (0.44–1.38)	
No	42.9 (151)	10.9 (23/211)	1	0.39
**Any current male/female condom use**				
Yes	79.6 (280)	10.4 (42/403)	1.72 (0.73–4.05)	
No	20.5 (72)	6.1 (6/98)	1	0.19
**Current use of oral contraceptive pills**				
Yes	13.4 (47)	15.4 (10/65)	1.80 (0.90–3.62)	
No	86.6 (305)	8.7 (38/436)	1	0.12
**Current use of injectable contraceptive**				
Yes	58.5 (206)	6.4 (19/296)	0.46 (0.26–0.81)	
No	41.5 (146)	14.2 (29/205)	1	0.007
**Contraception use during follow up (time varying)**				
**Consistent condom use**				
Yes	–*	8.5 (26/328)	0.60 (0.34–1.07)	0.09
No		12.0 (22/173)	1	
**Any current male/female condom use**				
Yes	–*	8.8 (33/375)	0.76 (0.41–1.41)	0.40
No		11.9 (15/126)	1	
**Use of oral contraceptive pills**				
Yes	–*	16.7 (13/78)	2.02 (1.07–3.83)	0.04
No		8.3 (35/423)	1	
**Use of injectable contraceptive**				
Yes	–*	6.6 (22/330)	0.41 (0.23–0.71)	0.002
No		15.5 (26/168)	1	

* Data from multiple time points contribute to this statistic, number for each time point not shown.

### Sample Characteristics

At screening, most women (58.5%) self-reported use of injectable contraception while 13.4% reported oral contraceptive pill use (Table 1). Regarding condom use, using the derived measure, 57.1% reported consistent condom use in the 6 months prior to screening while 79.6% reported current use of male or female condoms as measured by a single (yes/no) question. About a quarter (28.1%) of women reported heavy drinking, marijuana use, and/or use of these during sex or some combination of these activities (Table 2). Heavy drinking accounted for most of these high risk activities. Five percent of women reported a recent STI diagnosis and most women (74.4%) had a single a main sex partner at screening.

**Table 2:** HIV risk indicators* at screening by incident pregnancy.

	Column % (n)	Rate per 100 woman yrs (No. pregnancies/woman yrs)	Unadjusted Rate Ratio (95% CI)	P-value
***Overall pregnancy incidence***	(352)	9.6 (48/501)	–	–
**Marijuana, heavy drinking** Λ **and/or use of these during sex**				
≥ 2 such activities	9.9 (35)	18.3 (9/49)	2.02 (0.96–4.23)	0.15
1 such activity	18.2 (64)Λ	7.1 (7/99)	0.80 (0.35–1.81)	
None	71.9 (253)	9.1 (32/253)	1	
**Diagnosed with sexually transmitted infection**				
Yes	5.1 (18)	20.8 (5/24)	2.38 (0.94–6.01)	0.10
No	95.9 (334)	9.0 (43/477)	1	
**Type of sex partner(s)**				
No partner	14.2 (50)	10.5 (7/67)	1.13 (0.50–2.56)	0.99
Causal only	2.3 (8)	10.3 (1/10	1.12 (0.15–8.19)	
Main partner only	74.4 (262)	9.3 (35/375)	1	
Main & casual partners	9.1 (32)	10.0 (5/50)	1.07 (0.42–2.74)	
**Main sex partner >10 yrs older**				
Yes	8.8 (26)	7.8 (3/38)	0.79 (0.24–2.57)	0.68
No	91.2 (268)	9.6 (37/386)	1	
**Living situation with main partner**				
Lives with main partner	45.5 (160)	9.0 (21/233)	1	0.92
Does not live with main partner	38.1 (134)	9.9 (19/192)	1.09 (0.59–2.03)	
No main partner	16.5 (58)	10.5 (8/76)	1.17 (0.52–2.64)	
**Number of sexual partners**				
≥2 partners	15.6 (55)	11.5 (9/78)	1.25 (0.60–2.58)	0.56
1 partner	84.4 (297)	9.2 (39/423)	1	
**Has partner of unknown HIV status**				
≥1 partner(s)&	48.9 (172)	8.4 (20/239)	0.78 (0.44–1.38)	0.39
No such partners (knows status)	51.1 (180)	10.7 (28/262)	1	
**Partner type by knowledge of HIV Status** **				
>2 partners varied knowledge	9.5 (33)	6.2 (3/48)	1.04 (0.29–3.72)	0.05
2 partners both unknown status	6.3 (22)	20.0 (6/30)	3.42 (1.26–9.25)	
1 partner known negative	46.4 (162)	12.0 (28/234)	2.03 (1.01–4.07)	
1 partner unknown status	37.8 (132)	6.0 (11/184)	1	

* Refers to six months prior to screening interview.

Λ Heavy drinking defined as 5 or more alcoholic drinks in one day; 81% (52/64) of these women were heavy drinkers.

& Includes women with partners where HIV status was either all unknown, or status known for 1 of multiple partners.

** Excludes 3 women with known HIV-positive partners as data too scant for a separate stratum. No pregnancies occurred among these women.

### Pregnancy Rates and Outcomes by Vaccination Period

Of 51 total pregnancies, 48 first pregnancies occurred, or 13.6% (48/352) of women became pregnant. Overall pregnancy incidence was 9.6/100 w-yrs [48/501.1 w-yrs, 95% confidence interval (CI) 7.22 –12.71]. Pregnancy incidence during and after the vaccination period was 6.8 and 11.3/100 w-yrs, respectively; RR 0.60 (95%CI: 0.32–1.14; p = 0.10) (Table 3). Overall, half (47%) of pregnancies resulted in a full-term live birth. The distribution of other birth outcomes is shown in Table 3.

**Table 3:** Pregnancy incidence and outcomes by vaccination period.

Pregnancy rates	Outcome of pregnancy*			Row % (n)		
	Full-termlife birth	Prematurelife birth	Fetal death/still birth	Spontaneousabortion	Ectopic pregnancy	Electiveabortion
**Overall rate per 100 w-yrs**						
9.6 (48/501)**	47 (20)	16 (7)	2 (1)	7 (3)	2 (1)	26 (11)
**Rate during vaccination period** Λ						
6.8 (13/191)**	23 (3)	23 (3)	0	8 (1)	0	46 (6)
**Rate after vaccination period**						
11.3 (35/310)**	57 (17)	13 (4)	3 (1)	7 (2)	3 (1)	17 (5)

* Outcome of pregnancy not known for 5 women.

** Number of pregnancies/woman years.

Λ Defined as the period between a woman’s first scheduled vaccination through 1 month after the third, and final scheduled vaccination for that woman.

### Baseline Associations with Pregnancy –Unadjusted

Pregnancy rates were lower among women using injectable contraception at screening (hazard ratio [HR] 0.46, 95% CI 0.26 –0.81) (Table 1). Compared with women with a single partner of HIV-unknown status, pregnancy rates were increased both among women with a single partner known to be HIV-negative [HR = 2.03 (1.01–4.07)], and among women with 2 partners also of unknown HIV status [HR = 3.42 (1.26–9.25)] (Table 2). Pregnancy rates tended to be higher among women reporting sexually transmitted infections or a combination of two or more of the following factors: marijuana use, heavy drinking or use of these during sex, though confidence intervals were wide (Table 2).

### Time-varying Associations with Pregnancy – Unadjusted

Pregnancy rates were reduced among women who reported using injectable contraception during the trial [HR = 0.41 (0.23–0.71)] (Table 1). There was a similar protective trend among women reporting consistent condom use during the trial [HR = 0.60 (0.34–1.07)], but not among women reporting condom use when measured as a single, “yes/no” question [HR = 0.76 (0.41–1.41)]. Women reporting use of oral contraceptive pills (OCPs) during the trial had double the pregnancy incidence of those not reporting such use [HR = 2.02 (1.07–3.83)].

### Multivariable Associations with Pregnancy

Pregnancy rates were reduced among women enrolled at a trial site that provided hormonal contraception, who entered the study as consistent condom users or as users of injectable contraceptives (Table 4). Women who engaged in at least two different high risk substance use behaviours such as heavy drinking, marijuana smoking, or use of these substances during sex had double the pregnancy incidence compared with women who did none of these activities [HR = 2.66 (1.24–5.72)]. Compared with women reporting a single partner of unknown HIV status, pregnancy rates were increased both among women with a single partner but whose status was known to be HIV-negative [HR = 2.34 (1.16–4.73)] and among women with 2 partners also with unknown HIV status [HR = 4.42 (1.59–12.29)]. Considering all behavioural risk factors identified (inconsistent condom use, non-use of injectable contraceptives, and drug/alcohol use), pregnancy incidence was 7.1/100 w-yrs among women with none or only one such risk factor and was 17. 4/100 w-yrs among women with at least two of these risk factors, [HR = 2.46 (1.39 –4.35), p = 0.02].

**Table 4:** Multivariable model* of screening factors associated with incident pregnancy.

	Unadjusted Rate Ratio	AdjustedΛ Rate Ratio (95% CI)	p-value
**Contraception supplied at site**			
Yes	0.68	0.43 (0.22–0.86)	0.02
No	1	1	
**Consistent condom use** (in 6 months prior to screening)			
Yes	0.78	0.54 (0.28–1.04)	0.07
No	1	1	
**Using injectable contraception at screening**			
Yes	0.46	0.37 (0.21–0.67)	0.0009
No	1	1	
**Marijuana use, heavy drinking** ** **and/or use during sex** (in 6 months prior to screening)			
≥ 2 such activities	2.02	2.66 (1.24–5.72)	0.05
1 such activity	0.80	0.85 (0.36–2.02)	
None	1	1	
**Partner type by knowledge of HIV status** (in 6 months. prior to screening)			
>2 partners mixed knowledge of status	1.04	1.01 (0.27–3.74)	0.01
2 partners both unknown status	3.42	4.42 (1.59–12.29)	
1 partner known negative	2.03	2.34 (1.16–4.73)	
1 partner unknown status	1	1	

* Final model does not include 3 women with known HIV-positive partners as this stratum was too thin for modeling.

Λ Adjusted for all other variables in the model; for variables with >2 levels the overall p value shown.

** Heavy drinking defined as 5 or more alcoholic drinks in one day.

## Discussion

Overall the pregnancy rate was at the low end of the range observed in other HIV prevention trials, on par with the one other vaccine trial reporting on pregnancy incidence in sub-Saharan Africa [10], and was half of that seen among African women enrolled in non-trial cohorts [16], [17]. Women in this trial were largely able to adhere to their initial agreement to avoid becoming pregnant during the vaccination period. Although the difference in pregnancy rates during and after the vaccination period was not statistically significant, there was a trend to higher rates after the vaccination period. We expect that the true difference is even greater since pregnancy testing in the post-vaccination period was less rigorous and likely under estimated the true pregnancy rate.

The proportion of elective abortions appeared to be elevated for pregnancies conceived during the vaccination period, compared with those conceived later, although we cannot conclude this with certainty because the study was not powered to evaluate this finding. We do not have information on women’s motivations, but a range of explanations for these elective terminations are possible. These may include: more immediate access to the health care system during trial participation; that women perceived it was unsafe to conceive a pregnancy during the vaccination period as they were advised to be on contraception during this time; or may simply reflect that pregnancies in the post-vaccination period were more often intended given women’s agreement to avoid them during the vaccination period. We lack detail on the circumstances of the elective abortions, but this procedure is legal in South Africa. Regarding pregnancy outcomes, those observed here were on par with another trial [7] and from a representative community sample of South African women [18].

Encouraging was that predictive factors were identified, over-and-above the requirement that women use at least two forms of contraception as was required in this trial. All of the additional factors can be readily measured during screening for a large clinical trial, some are modifiable at trial entry, and preventive measures were associated with at least a halving in pregnancy risk. It is likely that the convenience, immediacy, certainty, and quality-of-care associated with on-site injectable contraceptive access is critical to its preventive role, as this finding is buttressed by another trial showing that women obtaining contraception off-site were at increased pregnancy risk [10]. The pregnancy rates among injectable contraception users was higher than that typically seen for “perfect,” established users. It may reflect imperfect measurement as these findings were based on self-report for an unspecified timeframe of “current use” not clinical records, and thus may not have adequately captured the exact duration of use. Alternatively, women may not have been established users.

We also found that women who entered the trial using, and who continued to use, injectable contraceptives, but not oral contraceptive pills, were less likely to get pregnant. Despite being counter-intuitive, this finding has also been observed elsewhere: in a South African microbicide trial, pregnancy rates were 11.5/100 w-yrs among oral contraceptive users and <2.0/100 w-yrs among women on injectables [13]. Even though both injectables and the pill have high method-effectiveness, women’s ability to consistently and/or correctly use the pill may diminish its method-effectiveness, and result in lower use-effectiveness, which takes into account user error. It is not biologically possible for oral contraceptives to increase pregnancy risk, and there are several possible explanations for observing a positive association between oral contraceptives and pregnancy. First, some women reporting oral contraceptive use, may not have been using a legitimate contraceptive, as there are pills in the marketplace that may not be contraceptive, but are advertised or understood as such. Second, as some form of hormonal contraception was required during the trial, those not using oral contraceptives were therefore using injectables, and the contrast between these two groups may simply reflect the greater use-effectiveness of injectables. Alternatively, when faced with the hormonal-method requirement, those opting for oral contraceptives may reflect a group of women: ambivalent about their pregnancy desires; not fully committed to avoiding pregnancy who thus opted for a self-dosing method, or; committed to avoiding pregnancy but who had difficulty adhering to the self-dosing regimen required by oral contraceptives.

Regarding condoms, how use is measured is important for distinguishing between casual and consistent users, as we found that only the latter group was at lowered pregnancy risk. Condom use measured via a single, “yes/no” question about “current” use, was not predictive, while consistent condom use, derived from a series of questions enquiring about condom use by each partner type during a specified timeframe, showed a trend toward halving pregnancy risk in the adjusted analysis. Careful measurements of condom use to determine consistent use has also been shown as protective by Reid et al., and use at last sex before study entry (but as not as one’s main method) by Halpern et al. in trial settings. Together these findings suggest that condoms may be a viable method for pregnancy prevention, so long as they are used consistently or recently. In contrast, if only a single, non-specific condom use measure is employed and women say “yes” to current condom use without further corroboration, (either in response to additional questioning or by failing to demonstrate familiarity with condoms when using a model) these women should be flagged as those in need of more intensive pregnancy counselling and support.

Pregnancy was also a function of HIV status, and number, of partners. Using women with a single partner of unknown HIV status as a reference, we were able to examine the unique impact of HIV status alone (by comparing pregnancy rates of women with only one partner with unknown status vs. negative HIV status) and of multiple partners (by comparing the impact of 1 versus 2 partners among women who didn’t know their partners’ HIV status). Regarding the former, knowing a sex partner’s HIV status may be emerging as a partnership factor of interest. Not knowing a partner’s HIV status may be a marker of a newer relationship where HIV status has not yet been discussed and so women were taking greater precautions against becoming pregnant. Alternatively, it may reflect a relationship where HIV status cannot be discussed, and thus women were hesitant to cement the relationship further with a pregnancy. We were unable to test these hypotheses as we lacked data on relationship duration and disclosure. This finding deserves further research. Regarding multiple partners, this may be a marker for women with more risky behaviour in general, as multiple partnering is also a risk factor for HIV. It is unclear whether the multiple partnering seen here is due to commercial transactions, as almost no women reported engaging in commercial sex when specifically asked (data not shown as it was extremely uncommon).

While almost twenty percent of the sample reported heavy drinking, it was heavy drinking along with either marijuana use, or concurrent drinking and sex that were associated with a two fold risk of pregnancy. Heavy drinking and recreational drug use are well-established risk factor for HIV in South Africa [19]. The distinct nature of our finding requires corroboration, but preliminary implications for clinical trialists are that women reporting multiple risk factors, but not heavy drinking alone, should be flagged for increased pregnancy prevention counselling, and may be especially suitable candidates for trials given their increased risk for HIV, and their need for the risk reduction packages offered within trials. However, the larger public health problem of alcohol use and pregnancy remains.

### Strengths and Limitations

Strengths of this analysis are that it is the first report from a vaccine trial on risk factors for pregnancy and the longitudinal design with high retention enhanced ability to make causal inferences. Factors examined were all collected within the context of a typical clinical trial, and didn’t require specialized interviewing techniques, yet were highly predictive of pregnancy risk. This bodes well for future trials as women can be readily screened for pregnancy using a few questions. Limitations of this analysis were the variable pregnancy prevention messages and pregnancy outcome ascertainment once the trial was interrupted. Nonetheless, we were still able to make valid comparisons within the dataset as the direction of the bias was known, but given the lack of systematic pregnancy testing in the post-vaccination period, overall pregnancy rates may be underestimated.

### Conclusion

It is possible to efficiently screen women for pregnancy risk, and concrete steps such as providing injectable hormonal contraception for free on-site, and supporting consistent condom users, can reduce pregnancy risk among South African women in HIV trials. Additionally, among women with a single partner, differential knowledge of male partners’ HIV status impacts pregnancy rates and is a new finding that deserves further research to illuminate the underlying reason for its association with pregnancy. Given long-standing calls to better integrate family planning and HIV/STI risk reduction counselling, clinical trialists and health counsellors should make it a point to enquire about the number of, and male partner’s HIV status as a potential modifier of pregnancy and HIV risk. Together, these few simple steps may help to maximize the safety of the mother and children conceived during HIV prevention trials.
